# Reappraisal Modulates Attentional Bias to Angry Faces

**DOI:** 10.3389/fpsyg.2016.01841

**Published:** 2016-11-22

**Authors:** Shin Ah Kim, Hackjin Kim, Sang Hee Kim

**Affiliations:** ^1^Department of Brain and Cognitive Engineering, Korea UniversitySeoul, South Korea; ^2^Department of Psychology, Korea UniversitySeoul, South Korea

**Keywords:** attentional bias, orienting, disengagement, reappraisal, suppression, angry face, happy face

## Abstract

Heightened attentional bias to emotional information is one of the main characteristics of disorders related to emotion dysregulation such as anxiety, depression, and substance abuse. Although reappraisal, an emotion regulation strategy, is known to effectively modulate subjective experience of emotions, it remains unknown whether reappraisal can alter attentional biases to emotional information. In the current research, we investigated the influence of instruction-induced state reappraisal (Study 1) and trait reappraisal (Study 2) on attentional biases to happy and angry faces. In Study 1, healthy young women were recruited and randomly assigned to one of the three groups: up-, down-, and no-regulation. Participants were instructed to reappraise their emotions to increase and decrease emotional experience while viewing an emotionally negative film clip. Attentional bias was assessed with a dot-probe task with pictures of angry and happy facial expressions. In Study 2, a separate group of healthy young men and women participated. Participants’ trait reappraisal and suppression as well as state and trait anxiety were assessed. A dot-probe task was completed by all participants. Statistical tests in Study 1 revealed that participants who reappraised to decrease negative emotions while viewing an emotionally negative film clip had reduced attentional bias to subsequently presented angry faces compared to participants who reappraised to increase negative emotions. Multiple regression analyses in Study 2 revealed that trait reappraisal predicted slower orienting toward angry faces, whereas state anxiety predicted slower disengagement from angry faces. Interestingly, trait suppression predicted slower disengagement from happy faces. Taken together, these results suggest that both instruction-induced state reappraisal and trait reappraisal are linked to reduced attentional bias to negative information and contribute to better understanding of how everyday emotion regulation styles contribute to attentional processing of emotional information.

## Introduction

Individuals have a biased tendency to attend more to emotionally meaningful versus ordinary information (Mathews and MacLeod, 1994; Todd et al., 2012). Although an attentional bias to emotional information is helpful and facilitates adaptive responses to environmental challenges (Lang et al., 1997; Öhman et al., 2000), regulating task-irrelevant attention to emotional information is necessary for psychological and physical well-being (Milberger et al., 1997; Shaw et al., 2014).

In fact, dysfunctional attentional biases to emotional information have been linked to various emotion dysregulation disorders including anxiety, depression, and substance abuse. For example, patients with anxiety show biased attention to threat-related information, and substance abusers also attentional biases to substance-related information (MacLeod et al., 1986; Cox et al., 2002). Attentional biases to task-irrelevant emotional information may play a critical role in the development and maintenance of emotional disorders (Pergamin-Hight et al., 2015). Heightened anxiety and stress reactivity were observed in healthy individuals when an attentional bias to threat-related information was experimentally induced (Mathews and MacLeod, 2002; Eldar et al., 2008). In contrast, training attentional avoidance of negative information led to reduced state anxiety (Amir et al., 2008) and reduced emotional reactivity to stressors (Dandeneau and Baldwin, 2009).

Given the critical association between emotion dysregulation and increased attentional bias (Bradley et al., 1997; Todd et al., 2012), one may wonder whether emotion regulation training could reduce task-irrelevant attention to emotional information. A substantial number of studies on emotion regulation have shown that voluntarily changing the emotional meaning of presented information (i.e., reappraisal; Gross, 2002) alters subjective and neural correlates of emotions (Ochsner et al., 2002; Kim and Hamann, 2007). Surprisingly, however, little research has investigated whether cognitive reappraisal can change attentional bias to emotional information. One study with subclinical smokers revealed that those who reappraised smoking cravings showed reduced attentional bias and diminished craving toward smoking related cues compared with those who suppressed and accepted smoking cravings (Szasz et al., 2012). However, whether reappraisal can modulate attentional bias in healthy individuals remains unknown.

In addition, the momentary effect of reappraisal may accumulate over time if reappraisal is habitually used for daily emotional events, and this could produce a systematic difference in attentional responses to emotional information. Indeed, individual differences in trait-level reappraisal have been associated with daily positive and negative emotional experiences (Gross and John, 2003). Neural evidence indicated that individuals with greater reappraisal scores tended to show decreased amygdala activity and increased medial prefrontal activity in response to angry and fearful faces (Drabant et al., 2009; Nelson et al., 2015). A previous neuroimaging study with a dot probe task revealed increased activity in the left amygdala as participants responded faster to the fearful faces presented on their left visual field (Carlson et al., 2009). Therefore, given a habitual tendency to use reappraisal associated with decreased amygdala activity, one can predict that reappraisal would be associated with slower orienting responses to emotional information.

In the current research, we investigated the effects of momentary, instruction-induced reappraisal and habitual reappraisal on attentional biases in two studies. Attentional bias was assessed with a dot-probe task (MacLeod et al., 1986), which is widely used to assess attentional biases. In the dot-probe task, stimuli with different emotional meanings are presented briefly, and a dot probe appears in the location where an emotional or neutral stimulus was presented. Individuals are faster at detecting probes that appear in the same location as emotional versus neutral stimuli. This relative response time difference provides an index of attentional bias, wherein a greater difference indicates a stronger tendency to attend to emotionally salient over less emotionally salient information. Attentional bias can also be divided into two independent subcomponents: more rapidly allocating attention to, and more slowly disengaging from, emotional information (Koster et al., 2004). It has been proposed that attentional orienting that appears in the early stages of attentional processing is more likely to be dependent on automatic and involuntary processes, whereas attentional disengagement that appears in the later stages of attentional processing is more likely to be dependent on the observer’s intention and voluntary processes (Theeuwes, 2010; Pool et al., 2016).

In Study 1, we investigated the effect of instruction-induced reappraisal on attentional bias to subsequently presented faces. Healthy young women were recruited and randomly assigned to one of the three reappraisal groups: up-regulation, down-regulation, and no-regulation. First, emotional states were induced by having participants watch a film clip showing a female victim of physical violence. While watching the film, participants were instructed to use reappraisal to increase or decrease emotions, or did not reappraise their emotions. This was followed by a dot-probe task with angry, happy and neutral faces. For Study 1, only women participants were recruited because naturally occurring initial emotional reactions to a female victim may differ across men and women. Our main hypothesis was that reappraisal to increase negative emotions would lead to a greater attentional bias to angry faces, whereas reappraisal to decrease negative emotions would lead to a reduced attentional bias to angry faces. We also examined attentional biases to happy faces. A previous study reported no changes in attentional bias to positive words after negative mood induction (Tamir and Robinson, 2007). As participants were instructed to reappraise emotions elicited by a film clip with negative emotional content, we expected no differences in attentional bias to positive faces across different reappraisal conditions.

In Study 2, we further tested whether habitual daily uses of reappraisal and suppression predict attentional biases to angry and happy faces. Healthy young men and women performed a dot-probe task as in Study 1. Individual differences in reappraisal and suppression were assessed with the Korean version of the Emotion Regulation Questionnaire (ERQ; Gross and John, 2003). State and trait anxiety were also assessed. Based on the available evidence (Carlson et al., 2009; Drabant et al., 2009; Nelson et al., 2015), we hypothesized that the habitual tendency to use reappraisal to regulate emotions would be associated with slower orienting toward emotional faces.

## Study 1

### Materials and Methods

#### Participants

Fifty-seven healthy young women (mean age = 22.30 ± 2.65 years) were recruited through campus flyers. The sample size was determined based on pilot studies and previous studies demonstrating the effect of reappraisal using between group design (Williams et al., 2009; Szasz et al., 2011). All volunteers were college students who reported no past or current diagnosis of neurological or psychiatric disorders. All participants were right-handed according to the Edinburgh Handedness Inventory (Oldfield, 1971). After the study procedures were explained, participants gave written informed consent and were randomly assigned to one of three groups: up-, down-, or no-regulation. This study was performed in accordance with the Declaration of Helsinki and the procedures were approved by the local institutional review board. Participants received monetary compensation for their time.

#### Stimuli and Dot-Probe Task

For the dot-probe task, we selected faces with angry (valence 1.82 ± 0.06; arousal 3.81 ± 0.12), happy (valence 4.14 ± 0.15; arousal 3.01 ± 0.07), and neutral (valence 2.74 ± 0.15; arousal 2.51 ± 0.10) expressions from a standardized database of face photographs (Lee et al., 2013). Valence and arousal ratings were based on a 5-point scale (1 = extremely unpleasant/not at all arousing, 5 = extremely pleasant/extremely arousing). Thirty-seven pairs of faces (21 female and 16 male) were used, consisting of 14 angry-neutral pairs, 14 happy–neutral, and 9 neutral–neutral pairs. Faces in each pair were from the same person. The dot-probe task was programmed using E-Prime (Psychology Software Tools Inc., Sharpsburg, PA, USA). Each trial started with a fixation point in the center of the screen for 500 ms. This was followed by a pair of faces, one on each side of the screen, for 500 ms. When the faces disappeared, a small gray dot appeared either on the left or right side of the screen. Participants were instructed to press one of two response keys on the computer keyboard (“z” or “/”) to indicate whether the dot was on the left or right as quickly and accurately as possible. Each of the 37 face pairs were shown four times, and were fully counterbalanced in terms of presentation side and facial expression. There were 148 trials. The task took approximately 8 min, including a 1-min break halfway through. During the break, participants were instructed to have a rest with their hands off the keyboard.

#### Procedure

After signing a consent form, participants completed the Beck Anxiety Inventory (BAI; Beck et al., 1988) and Emotion Regulation Questionnaire (ERQ; Gross and John, 2003). The ERQ assesses individual differences in the use of reappraisal versus expressive suppression in regulating emotions in daily life (Gross and John, 2003). Then, participants rated their current mood using four bipolar visual analog scales (VAS; drowsy-alert, relaxed-anxious, depressed-happy, and amicable-angry). Each VAS was 100 mm long and ranged a score from 0 to 100, yielding a total VAS scale sum of 400. Participants were asked to move the cursor from the center toward either extreme of the scale until cursor position represented their current mood.

Next, participants received emotion regulation instructions according to group assignment (Kim and Hamann, 2007). Participants in the up-regulation group were instructed to try to increase emotions that might be elicited by the film clip. In contrast, those in the down up-regulation group were instructed to try to decrease emotions that might be elicited by the film clip. Specifically, the up-regulation group was encouraged to imagine the scenes as more personally relevant or physically closer to them. Instead, the down-regulation group was encouraged to imagine the scenes as less personally relevant or physically distanced from them. Finally, participants in the no-regulation group were instructed not to regulate their emotions and to view the film naturally.

Next, participants watched a 5-min film segment selected from the movie “One on One (2014)” directed by Kim Ki-Duk. This film segment depicted a man’s violent behavior against his girlfriend. An independent group of 16 participants (nine men and seven women) rated the film segment as having an emotional valence of 2.1 (1 = negative, 7 = positive) and emotional arousal of 5.2 (1 = weak, 7 = strong). Immediately after watching the film, participants again rated their mood on the VAS (VAS-post). Participants also rated how successful they were in following the reappraisal instructions on a VAS (0 = not good at all, 100 = very successful). Finally, participants performed the dot-probe task. There were eight practice trials composed of face pairs that were not used in the main task before the experimental trials. After completing the task, participants were debriefed and compensated for their time.

#### Data Analysis

A total of 1.1% of the total data were excluded due to incorrect responses (0.4% of the data) and outlier responses, defined as trials with reaction times less than 200 ms or greater than three standard deviations above the individual mean (0.7% of the data). Attentional bias scores were calculated by subtracting mean reaction times to dot probes that replaced emotional faces from mean reaction times to dot probes that replaced neutral faces. Positive attentional bias scores indicate greater attention to emotional relative to neutral faces, and negative attentional bias scores indicate avoidance of emotional faces. Attentional bias was calculated separately for happy and angry faces. We further assessed two subcomponents of attentional bias: orienting and disengagement. Orienting was calculated by subtracting mean reaction times to probes replacing emotional faces in emotion-neutral pairs from mean reaction times to probes replacing neutral faces in neutral–neutral pairs. Greater positive scores indicate faster orienting to emotional faces. Disengagement was calculated by subtracting mean reaction times to probes replacing neutral faces in neutral-neutral pairs from mean reaction times to probes appearing in the location of the neutral expression in emotion-neutral pairs. Greater positive scores indicate slower disengagement from emotional expressions. Statistical analyses were performed using SPSS 19 (SPSS Inc., Chicago, IL, USA).

### Results

#### Participant Characteristics and Mood Change by Reappraisal

Descriptive statistics for each group are presented in **Table 1**. There were no groups differences in age [*F*(2,54) = 1.30, *p* = 0.282], reappraisal [*F*(2,54) = 0.82, *p* = 0.448], suppression [*F*(2,54) = 2.25, *p* = 0.116], and BAI [*F*(2,54) = 1.31, *p* = 0.278] scores (**Table 1**).

**Table 1:** Means and standard deviations for age, reappraisal, suppression, and BAI scores in down-regulation, no-regulation, and up-regulation groups.

Group	Down-regulation (*N* = 19)		No-regulation (*N* = 19)		Up-regulation (*N* = 19)		Statistics	
	Mean	*SD*	Mean	*SD*	Mean	*SD*	*F*	*p*
Age	22.95	3.04	22.37	2.73	21.58	2.01	1.30	0.282
ERQ								
Reappraisal	28.57	5.04	26.00	7.99	27.84	5.85	0.82	0.448
Suppression	13.00	3.90	12.89	3.35	10.74	3.87	2.25	0.116
BAI	7.84	5.98	9.89	7.36	6.68	4.99	1.31	0.278
VAS							12.82	<0.001
Pre	160.15	48.43	159.89	53.07	147.38	39.48	0.45	0.639
Post	218.89	33.44	269.01	42.60	309.08	38.57	26.33	<0.001

*ERQ, Emotion Regulation Questionnaire; BAI, Beck Anxiety Inventory; VAS, Visual Analog Scale.*

Negative mood scores were calculated by summing all VAS scores (depressed-happy scores were reverse-coded). A two-way ANOVA with time (pre versus post) as a within-subject factor and group as a between-subject factor was conducted. The main effects of group, [*F*(2,54) = 8.37, *p* = 0.001] and time [*F*(1,54) = 175.00, *p* < 0.001] and the group × time interaction, [*F*(2,54) = 12.82, *p* < 0.001], were significant. Overall, negative mood increased post-film [mean (M) = 265.66, standard deviation (*SD*) = 52.98] compared with pre-film (*M* = 155.81, *SD* = 46.87). Overall the up-regulation group reported greater negative mood (*M* = 228.23, *SD* = 29.55) compared with the down-regulation group (*M* = 189.52, *SD* = 29.55). To further examine the interaction effect, follow-up *t*-tests were conducted. Results showed no group differences in negative mood before the film (*t*s < 1). However, a significant group effect was found post-film, *F*(2,54) = 26.33, *p* < 0.001. The down-regulation group reported lower negative mood than the up- [*t*(36) = 7.70, *p* < 0.001] and no-regulation groups [*t*(36) = 4.03, *p* < 0.001]. The up-regulation group reported greater negative mood than the no-regulation group, [*t*(36) = 3.04, *p* = 0.004]. A one-way ANOVA on self-ratings of regulation success with group as a factor revealed no group differences, *F*(2,54) = 1.44, *p* = 0.246.

To specifically examine whether mood change after watching the film differed across groups, we calculated mood change scores by subtracting pre-film scores from post-film scores. A one-way ANOVA on mood change scores with group as a factor revealed a significant main effect [*F*(2,54) = 12.82, *p* < 0.001]. Follow-up *t*-tests showed that the increase in negative mood scores was greater in the up-regulation group than no-regulation group [*t*(36) = 2.42, *p* = 0.021] and down-regulation groups [*t*(36) = 5.92, *p* < 0.001]. The increase in negative mood was significantly smaller for the down-regulation group compared to no-regulation group [*t*(36) = 2.34, *p* = 0.025].

#### Attentional Bias and Reappraisal

To examine whether participants demonstrated reliable attentional bias to angry and happy versus neutral faces, we first conducted one-sample *t*-tests comparing attentional bias scores for positive and negative faces against zero. There was a significant bias to angry faces, *t*(56) = 4.87, *p* < 0.001, but not happy faces, *t*(56) = -1.32, *p* = 0.190. We then investigated how these attentional biases differed as a function of regulation, separately for angry and happy faces.

#### Angry Faces

**FIGURE 1:**
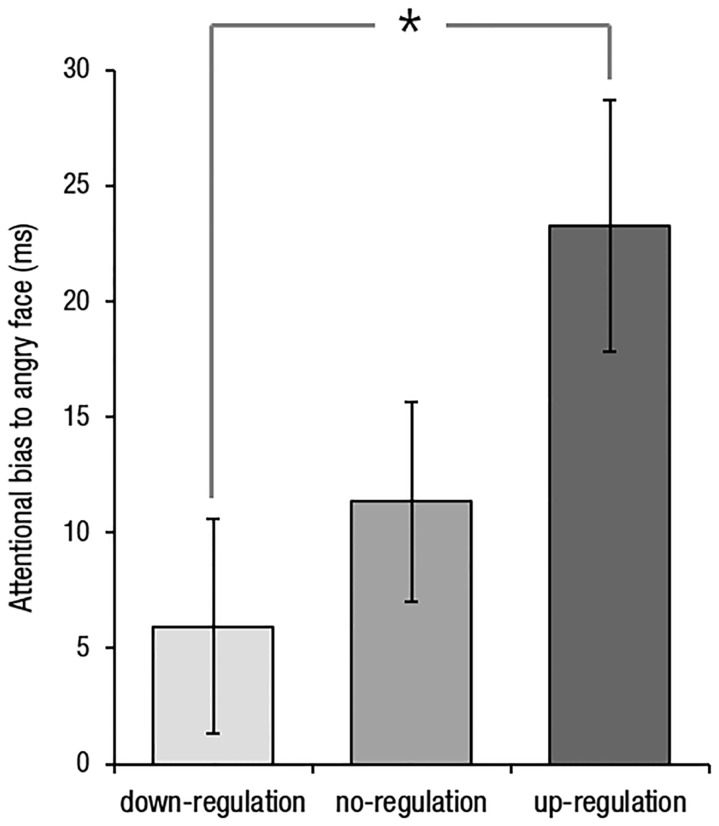
**Differences in attentional bias to angry faces between groups.** Error bars indicate standard error of the mean. The asterisk indicates statistical significance (*p* < 0.05).

A one-way ANOVA on attentional biases to angry faces with group as a factor revealed a significant effect of group, [*F*(2,54) = 3.38, *p* = 0.041]. Follow-up *t*-tests showed that the attentional bias to angry faces was reduced in the down-regulation group (*M* = 5.95, *SD* = 20.26) compared to the up-regulation group (*M* = 23.26, *SD* = 23.57), *t*(36) = 2.43, *p* = 0.020, but not to the no-regulation group (*M* = 11.34, *SD* = 18.88), *t*(36) = 0.85, *p* > 0.250. There was a marginally significant increase in attentional bias to angry faces in the up- compared to no-regulation group, *t*(36) = 1.72, *p* = 0.094 (**Figure 1**). A one-way ANOVA with group as a factor did not reveal any group differences in orienting to or disengagement from angry faces, *F*(2,54) = 0.80, *p* = 0.455, and *F*(2,54) = 2.20, *p* = 0.120, respectively.

#### Happy Faces

A one-way ANOVA on attentional biases to happy faces with group as a factor revealed no group differences, [*F*(2,54) = 0.10, *p* = 0.908]. No significant group effects were observed for orienting to happy faces, *F*(2,54) = 0.59, *p* = 0.556, and disengagement from them, *F*(2,54) = 1.15, *p* = 0.325.

## Study 2

### Materials and Methods

#### Participants

Forty-seven right-handed college students (22 male, mean age = 23.09 ± 2.10 years; 25 female, mean age = 22.06 ± 2.62 years) were recruited through campus flyers. The sample size was determined based on pilot studies and previous studies (Segerstrom, 2001; Koster et al., 2004). Participants reported no past or current diagnosis of neurological or psychiatric disorders. All participants provided written informed consent and received monetary compensation for participation. This study was performed in accordance with the Declaration of Helsinki and approved by the local Institutional Review Board.

#### Stimuli and Dot-Probe Task

The dot-probe task was similar to Study 1, except that the faces were selected from a different database and all conditions had the same number of trials. A total of 16 angry (valence 2.33 ± 0.41; arousal 4.84 ± 0.63), 16 happy (valence 5.93 ± 0.36; arousal 4.87 ± 0.47), and 64 neutral (valence 3.80 ± 0.23; arousal 2.63 ± 0.23) faces were selected from a standardized database of face photographs (Lee et al., 2006). Valence and arousal ratings were based on 7-point scales (1 = extremely unpleasant/not at all arousing, 7 = extremely pleasant/extremely arousing). Forty-eight pairs of faces were used, with 16 for each of the following combinations: happy–neutral, angry–neutral, and neutral–neutral. As in Study 1, faces in each pair were from the same person, but different pairs depicted different people. Trial sequence was the same as Study 1. There were 192 trials (48 face pairs repeated four times). The task took approximately 9 min (including a 1-min break).

#### Procedure

After signing the consent form, participants completed Spielberger State-Trait Anxiety Inventory (STAI; Spielberger, 1983). This scale includes a state (STAI-S) and a trait anxiety (STAI-T) subscale. Means and standard deviations for STAI-S and STAI-T were 45.55 ± 2.50 and 49.26 ± 3.12, respectively. Then, they received instructions regarding the dot-probe task and completed eight practice trials with faces that were not used in the actual task. Next, they completed the ERQ (Gross and John, 2003). Means and standard deviations for reappraisal and suppression were 28.53 ± 6.05 and 13.34 ± 4.13, respectively. The correlation between reappraisal and suppression scores was not significant (*r* = -0.19, *p* = 0.197).

#### Data Analysis

As in Study 1, trials with incorrect responses (0.7% of the data) and response outliers (1.2% of the data) were excluded from the analyses. Attentional bias, orienting, and disengagement scores were calculated separately for angry and happy faces. One-sample *t*-tests were performed comparing attentional bias scores against zero. We also performed multiple regression analysis to identify variables predictive for each angry and happy bias index (i.e., overall bias as well as orienting and disengagement). As predictor variables, we entered trait reappraisal, trait suppression, trait anxiety (STAI-T), state anxiety (STAI-S), as well as individual variables such as age and gender. Statistical analyses were conducted using SPSS 19 (SPSS Inc., Chicago, IL, USA).

### Results

**FIGURE 2:**
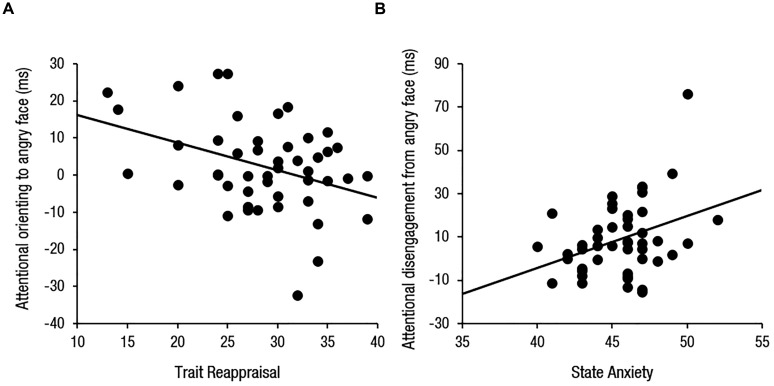
**Scatterplots illustrating relationships between (A)** trait reappraisal and attentional orienting and **(B)** state anxiety and attentional disengagement for angry faces.

**FIGURE 3:**
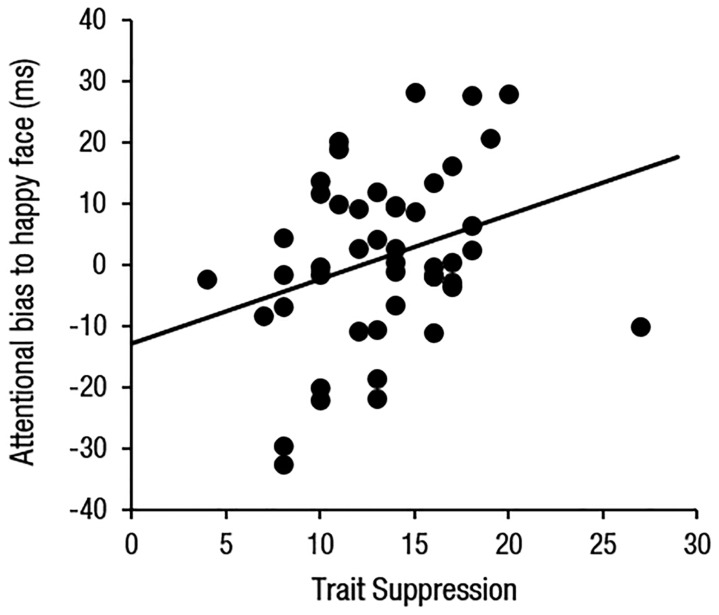
**A scatterplot illustrating the relationship between trait suppression and attentional bias for happy faces**.

One-sample *t*-tests revealed a statistically significant bias toward angry faces, *t*(46) = 3.89, *p* < 0.001, but no significant bias toward happy faces, *t*(46) < 1, *p* > 0.250, which is consistent with what we found from Study 1. Multiple regression analyses revealed that overall attentional bias toward angry faces were predicted by trait reappraisal (β = -0.32, *p* = 0.049) and state anxiety (β = 0.43, *p* = 0.007). Interestingly, reappraisal negatively predicted orienting to angry faces (β = -0.42, *p* = 0.012) and state anxiety positively predicted disengagement from angry faces (β = 0.43, *p* = 0.010; **Figure 2**). On the other hand, overall attentional bias toward happy faces was predicted by suppression (β = 0.40, *p* = 0.022; **Figure 3**). However, no predictive factors were observed for orienting. A marginal level of relationship between suppression and happy disengagement was found (β = 0.30, *p* = 0.086).

## Discussion

In this research, we investigated whether reappraisal modulated attentional biases to angry and happy faces in two studies. In Study 1, we found that self-reported negative mood, induced by a short film clip with unpleasant content, diminished in participants who reappraised to reduce negative emotions while watching the film clip as compared to those who reappraised to increase negative emotions and those who naturally viewed the film without reappraisal. More interestingly, participants who reappraised to reduce emotions also showed smaller attentional biases to subsequently presented angry faces compared to those who reappraised to increase emotions. There were no group differences in attentional biases to happy faces. In Study 2, we found that individuals more prone to using reappraisal in regulating everyday emotions were slower in orienting toward angry faces, whereas those more prone to using suppression were slower in disengaging from happy faces. Together, these results suggest that instructed and habitual patterns of emotion regulation are associated with attentional processes toward emotional information.

Across Studies 1 and 2, we found that both instruction-induced, state reappraisal and trait reappraisal contribute to attentional responses to angry faces. Reappraisal to reduce negative emotions during film viewing induced less vigilant attention to subsequently presented angry faces compared with reappraisal to increase negative emotions. Notably, participants were not instructed to reappraise emotions during the dot-probe task. Their explicit goal was to respond to the dot-probe as quickly as possible. Therefore, it appears that altered emotional mood states due to reappraisal while viewing the film clip were transferred to the experimental task and modulated attentional processes to subsequently presented angry faces. Alternatively, participants may have continued to reappraise during the dot-probe task, which could have directly influenced overt attention. However, a previous report showed that people tend to direct overt attention to the emotion source when they have an explicit reappraisal goal (Bebko et al., 2011). Therefore, we suggest that reappraisal-dependent changes in attentional biases to angry faces can be pronounced without explicit intention to regulate emotions and possibly through reappraisal-dependent changes in mood states.

Attentional bias did not differ between the down- and no-regulation groups. Therefore, it remains unclear whether this reduced attentional bias was truly due to decreased attentional bias in the down-regulation group or increased attentional bias in the up-regulation group. There was a trend toward a significant increase in attentional bias in the up- versus no-regulation group. Although the interpretation of these results is limited, the current findings still have important implications for clinical applications. Given that patients with emotional disorders have heightened attentional biases to task-irrelevant emotional information, our findings suggest that reappraisal may be useful for reducing a maladaptive level of attentional bias. More studies are needed to precisely elucidate the role of appraisal on attentional bias.

It has been repeatedly demonstrated that reappraisal has acute effects on affective states; however, its long-term cumulative effect still has to be determined (Adam et al., 2014; Ahn et al., 2015). The results from Study 2 provide helpful insight. Multiple regression analyses revealed that overall attentional biases to angry faces were predicted by trait reappraisal and state anxiety. More specifically, trait reappraisal contributed to the bias by slowing initial orienting toward angry faces, and yet, state anxiety contributed to the bias by slowing disengagement from angry faces. These results are partly consistent with previous results showing that high state-anxious individuals were slower to disengage from threatening words and faces relative to low state-anxious individuals (Fox et al., 2001). Interestingly, a previous study compared attentional biases to angry faces between individuals with stronger habitual reappraisal versus stronger habitual suppression (Arndt and Fujiwara, 2012). Results indicated that attentional bias was greater in reappraisers than suppressers, and this increase was due to slower disengagement from angry faces, which implies that reappraisers attend to angry faces longer than suppressors. At first glance, this seems partially contradictory to our finding of reduced attentional orienting for frequent reappraisers. However, if reappraisers generally direct overt attention to emotional information more than suppressors (Adam et al., 2014), the previous study may suggest that once attention is directed to negative information, reappraisers spend more time processing it than suppressors, allowing more elaborate cognitive processing of negative information even if it is task-irrelevant. On the contrary, our finding indicates that frequent reappraisers are less likely to direct attention to task-irrelevant negative information. Therefore, our results help clarify this small literature. That is, reappraisal appears to decrease detection sensitivity for negative information, but once that information is attended, more elaborate processing may take place to facilitate regulatory goal attainment. Together, results from the current and previous study suggest that reappraisal is associated with less sensitive orienting to and more prolonged and elaborate processing of negative information. This could play a key role in effective emotion regulation (Gross and John, 2003).

Interestingly, multiple regression analyses in Study 2 revealed that attentional biases to happy faces were predicted by trait suppression. More specifically, individuals with greater tendency to suppress emotions tended to disengage more slowly from happy faces even if they were task-irrelevant. However, the underlying mechanisms of the association between habitual suppression of emotions and attentional bias toward happy faces are still unclear. Although it remains speculative, longer attention time to dwell on happy faces signaling acceptance and safety might be instrumental in accomplishing an internally driven goal of managing emotions. Habitual suppression has been associated with heightened anxiety and increased sympathetic activation (Gross, 1998; Campbell-Sills et al., 2006), which could drive the regulatory goal. This speculation is consistent with the suggestion that difficulty in disengaging one’s attention is more likely to be influenced by the observer’s internal concerns or goals (Theeuwes, 2010; Pool et al., 2016).

It is notable that no measurable overall attentional bias to happy faces was observed. This lack of attentional bias to positive expressions is interesting given that quick and elaborate processing of positive information is as important and evolutionarily adaptive as quick and elaborate processing of negative information (Tobby and Cosmides, 1990). A previous study in which eye movements were monitored while participants viewed pairs of pictures depicting emotionally pleasant, unpleasant, or neutral scenes found that participants tended to attend to both pleasant and unpleasant pictures first compared to neutral pictures, and to attend to them for longer (Calvo and Lang, 2004). However, several previous studies have also reported absent or reduced attentional biases for positive stimuli (Fox et al., 2000; Öhman et al., 2001). Differences in emotional stimuli (faces versus events; happy versus affectionate), task (perceptual versus judgmental), and participants (typical versus patients) may contribute to these inconsistent results. A recent meta-analysis study of attentional bias toward positive emotional stimuli revealed larger attentional biases for babies, erotic attractive adults, and money, which are typically reported as highly arousing as compared with smiling happy faces (Pool et al., 2016). More research is needed to understand attentional biases to positive information.

Although the results of the current research are revealing, several limitations are also identified. First, in Study 1 we used a short film clip depicting a female victim of violence to induce negative emotions and found differences in attentional biases to angry faces following reappraisal. However, we did not observe differences in happy faces. It would be interesting and worthwhile to see whether reappraisal of positive emotions would result in changes in attentional biases to happy faces. Second, Study 1 tested only female participants in order to control sources of variability such as initial emotional responses to women victim and types of reappraisal strategies. A bigger scale of study with both male and female participants is warranted for better understanding of factors influencing attentional biases to emotional information. Third, in Study 1 we did not assess initial baseline levels of attentional biases before the exposure to the film clip. Repeated assessment of attentional biases before and after treatment may have the potential to increase demand characteristics bias. Despite that understanding, a baseline level of attentional bias measure for each subject would have allowed to draw stronger conclusions in relation to the influence of instructed reappraisal on attentional biases. Finally, in Study 2 we did not evaluate current mood states although we assessed current state anxiety. The inclusion of both current positive and negative affect as predictive factors in our analyses might have been more revealing, especially regarding the relationship between positive affect and attentional biases to happy faces.

To conclude, the main finding of the current research revealed that both instruction-induced state reappraisal and trait reappraisal are linked to reduced attentional bias and slower orientating to task-irrelevant angry faces. These results contribute to our understanding of how reappraisal regulates negative emotions and provides insight relevant to the development of treatment strategies for emotional disorders.

## Author Contributions

SAK and SHK conceived and designed the study. SAK conducted the study and collected the data. SAK and SHK analyzed the data. SAK, HK, and SHK interpreted the data. SAK drafted the first version of the manuscript. HK and SHK revised the manuscript. All authors approved the final version of the manuscript.

## Conflict of Interest Statement

The authors declare that the research was conducted in the absence of any commercial or financial relationships that could be construed as a potential conflict of interest.
